# Neoadjuvant Chemotherapy for Intraductal Papillary Mucinous Neoplasm-derived Pancreatic Cancer

**DOI:** 10.1097/SLA.0000000000006687

**Published:** 2025-03-05

**Authors:** Joseph R. Habib, Ingmar F. Rompen, Ammar A. Javed, Brady A. Campbell, Benedict Kinny-Köster, Po Hong Tan, Richard M. Miller, Riccardo Pellegrini, Alessio Marchetti, Paul C.M. Andel, Giampaolo Perri, Kelly J. Lafaro, D. Brock Hewitt, Jörg Kaiser, Lois A. Daamen, Thomas Hank, Greg D. Sacks, Adrian T. Billeter, Katherine Morgan, Oliver R. Busch, Beat P. Müller-Stich, Giovanni Marchegiani, Zhi Ven Fong, I. Quintus Molenaar, Marc G. Besselink, Markus W. Büchler, Christopher L. Wolfgang, Jin He, Martin Loos

**Affiliations:** * Department of Surgery, New York University Langone Health, New York, NY; † Department of Surgery, Regional Academic Cancer Center Utrecht, UMC Utrecht Cancer Center & St. Antonius Hospital Nieuwegein, Utrecht, The Netherlands; ‡ Department of General, Visceral, and Transplantation Surgery, Heidelberg University Hospital, Heidelberg, Germany; § Department of Surgery, Amsterdam UMC, University of Amsterdam, Amsterdam, the Netherlands; ∥ Cancer Center Amsterdam, Amsterdam, The Netherlands; ¶ Department of Surgery, Johns Hopkins Hospital, Baltimore, MD; # Department of Surgery, Mayo Clinic Arizona, Pheonix, AZ; ** Department of Surgery, Padua University, Padua, Italy; †† Division of Imaging and Oncology, University Medical Center Utrecht, Utrecht, The Netherlands; ‡‡ Clarunis University Center for Gastrointestinal Diseases, University of Basel, Basel, Switzerland; §§ Department of Pancreatic Surgery, Champalimaud Foundation, Lisbon, Portugal

**Keywords:** intraductal papillary mucinous neoplasm, pancreatic neoplasms, pancreatic cyst, pancreatic cancer, neoadjuvant therapy

## Abstract

**Background:**

Intraductal papillary mucinous neoplasm (IPMN)-derived pancreatic cancer is typically managed like pancreatic intraepithelial neoplasia–derived pancreatic cancer. However, in IPMN-derived pancreatic cancer, the role of chemotherapy remains controversial, particularly in the neoadjuvant setting (NAT).

**Objective:**

To evaluate the role of neoadjuvant chemotherapy in IPMN-derived pancreatic cancer.

**Methods:**

Patients with IPMN-derived pancreatic cancer treated with either upfront surgery (US) or NAT were identified from 8 international centers (2000–2023). Clinicopathologic data were compared. The date of the first treatment was used for Kaplan-Meier and log-rank tests to compare overall (OS) and recurrence-free survival. Multivariable Cox regression was performed in patients who underwent NAT.

**Results:**

In 1019 patients, 76 (7%) underwent NAT. Patients who received NAT had higher baseline CA19-9 levels (*P*<0.001). Of these 76 patients, 27 (36%), 20 (26%), and 29 (38%) had resectable, borderline resectable, or locally advanced pancreatic cancer at diagnosis, respectively. The advanced resectability stage was significantly more common in the NAT patients as compared with those who underwent US (*P*<0.001). OS for US patients was 38.0 months (95% CI: 33.7.1–44.3), which was not statistically different than those that received NAT [27.5 mo (95% CI: 23.1–46.7), *P*=0.121]. This was also valid for patients with resectable disease (US: 38.1 mo vs NAT: 35.6 mo, *P*=0.920). Complete or marked pathologic treatment response (*P*=0.046) and serological CA19-9 normalization after NAT (*P*=0.017) were associated with improved survival. On Cox-regression for OS, N2 disease [HR: 4.15 (95% CI: 1.71–10.10)], elevated CA19-9 [HR: 2.02 (95% CI: 1.06–3.85)] and R1 margin [HR: 2.36 (95% CI: 1.20–4.61)] was independently associated with OS after NAT, while resectability status was not.

**Conclusions:**

After NAT and resection, the advanced resectability stage was not associated with worse OS, indicating the value of this approach for borderline resectable and locally advanced IPMN-derived pancreatic cancer. The benefit of NAT in resectable disease is unclear and may require an individualized approach. Biological treatment effects can be assessed with CA19-9 and confirmed by pathologic response.

Intraductal papillary mucinous neoplasms (IPMN), the most common type of pancreatic cystic neoplasm, are being frequently identified due to the widespread utilization of cross-sectional imaging.^1^ These neoplasms are at risk of malignant degeneration and can develop into IPMN-derived pancreatic cancers.^2^ Conventionally, IPMN-derived pancreatic cancer is staged and managed based on extrapolated evidence established for the more common pancreatic intraepithelial neoplasm (PanIN)-derived pancreatic cancer. However, multiple studies have demonstrated superior outcomes in IPMN-derived pancreatic cancer.^3,4^ In support, these cancers are now believed to be biologically distinct entities.^5^

Accordingly, the benefit of chemotherapy for patients with IPMN-derived pancreatic cancer remains controversial.^6–8^ Recent evidence has suggested that patients with aggressive disease (elevated CA19-9 and nodal positivity) have an associated benefit from adjuvant chemotherapy, while those with less aggressive disease may not have this benefit.^7^ However, prospective evidence for this assumption is still lacking.

Beyond the use of adjuvant chemotherapy, the widespread implementation of neoadjuvant chemotherapy has been gaining momentum for PanIN-derived pancreatic cancer, particularly for patients with locally advanced (LAPC) or borderline resectable pancreatic cancer (BRPC).^9,10^ However, reports on the use of neoadjuvant chemotherapy in IPMN-derived pancreatic cancers are extremely scarce.^11^ The aim of the current study was to leverage the experiences of multiple high-volume pancreatic surgery centers and report on the observed impact, outcomes, and potential benefit of neoadjuvant chemotherapy for resected IPMN-derived pancreatic cancer.

## METHODS

### Study Population

Patients with IPMN-derived pancreatic cancer after pancreatic resection ranging between 2000 and 2023 were identified from institutional databases at eight international centers, including the University of Amsterdam, University Hospital of Basel, Heidelberg University, Johns Hopkins Hospital, Mayo Clinic Arizona, New York University, University of Padua, and the Regional Academic Cancer Center Utrecht. Patients with concomitant PanIN-derived pancreatic cancer separate from an adjacent IPMN, gross positive resection margin, metastatic disease at diagnosis, and 90-day postoperative mortality were excluded. Approval from local institutional review boards was obtained by each center. The strengthening and reporting of observational studies in epidemiology guidelines were implemented, and the study complied with the 1964 Helsinki Declaration and its corresponding amendments.^12^

### Pathologic Assessment and Definitions

Pathology was carefully reviewed by institution-specific pancreas specialized pathologists to verify that the invasive carcinoma and treatment effect was arising from an IPMN and not a separate concomitant PDAC. A tissue diagnosis of invasive cancer was obtained in all patients who received NAT before systemic therapy. Respectability was defined based on the National Comprehensive Cancer Network (NCCN) guidelines.^13^ The American Joint Committee on Cancer TNM system eighth edition was used for staging.^14–16^ An R1 resection margin was defined as microscopic evidence of tumor cells at or within 1 mm of the margin. The College of American Pathologists (CAP) tumor regression score was used to classify pathologic response to neoadjuvant chemotherapy (CAP data available from 5 of the participating centers).^17^

The date of the first treatment was used as time zero for all time-to-event analyses for overall survival (OS). This was the date of neoadjuvant chemotherapy initiation for the neoadjuvant chemotherapy (NAT) cohort or the date of surgery for the upfront surgery cohort. The pre- and post-neoadjuvant carbohydrate antigen 19-9 (CA19-9) values were used to group patients into 3 cohorts: (1) nonsecretors: CA19-9 <5 u/mL, (2) favorable CA19-9: normal (CA19-9 <37 both pre- and post-NAT) or normalizers (pre-NAT CA19-9 ≥37 u/mL and post-NAT CA19-9 <37), and (3) unfavorable CA19-9 (all patients with post-NAT ≥37).

### Statistical Analysis

Baseline characteristics and clinicopathologic data for patients who were treated with NAT versus upfront surgery were compared. Continuous variables, displayed as mean ± SD or medians and inter-quartile range (IQR), were compared by *t* test or Mann-Whitney *U* test. Categorical variables, displayed as frequencies and percentages, were compared using a χ^2^ test. Kaplan-Meier analysis with corresponding risk tables was employed for all time-to-event analyses, and these were used to derive median OS and recurrence-free survival (RFS). RFS was calculated from the time of surgery to recurrence, whichever occurred first. Log-rank tests were used to compare unadjusted and adjusted Kaplan-Meier curves. Patterns of first recurrence between NAT and upfront surgery were described and compared by χ^2^ tests. A subgroup analysis was performed in patients who received any chemotherapy, comparing survival after NAT to patients that received adjuvant chemotherapy only (ie, excluding patients that underwent surgery without any chemotherapy) and on the NAT cohort where OS was compared among CA19-9 responders and nonresponders in addition to major pathologic responders (CAP 0/1) and poor pathologic responders (CAP 2/3). Next, a proportional hazard Cox regression analysis was performed to assess factors associated with OS after NAT. Corresponding adjusted hazard ratios (HR) and 95% CI were reported. Multiple imputation was used to account for missing variables at random in the multivariable analysis. A C-index was used to evaluate the regressions. All other analyses were performed as a complete case analysis with missing variables provided in the corresponding tables. A *P* value of <0.05 was used to define statistical significance for all analyses. Statistical analysis was performed using the “R” statistical software (version 4.2.3).

## RESULTS

### Baseline Characteristics and Clinicopathologic Data: NAT Versus Upfront Surgery

Of the 1019 included patients, 76 (7%) underwent neoadjuvant chemotherapy followed by resection. FOLFIRINOX was the first-line treatment in most patients (n=42, 58%), while 30 patients received Gemcitabine-based regimens (40%). The median number of NAT cycles for all patients was 5 (IQR 4-8). Nine (12%) patients underwent neoadjuvant radiation therapy along with their neoadjuvant chemotherapy. Of the 943 patients who underwent upfront surgery, 933 (99%) had resectable disease. This was significantly more compared with those that underwent NAT, where 27 (36%), 20 (26%), and 29 (38%) patients had resectable, BRPC, or LAPC, respectively (*P*<0.001). Consequently, vascular resections were performed more frequently in the NAT cohort compared with the upfront surgery cohort (54% vs 17%, *P*<0.001). Median CA19-9 at diagnosis was 59 U/mL (IQR: 18–292) for patients undergoing upfront surgery versus 236 U/mL (IQR: 50–693) undergoing NAT (*P*<0.001). There were no other significant preoperative differences were observed between the 2 cohorts (Table 1).

**TABLE 1:** Baseline and Clinicopathologic Information Stratified by Patients Who Underwent Upfront Surgery Versus Neoadjuvant Chemotherapy

Variable	All patients N=1019	Upfront surgery N=943	Neoadjuvant N=76	*P*
Age, median (IQR)	70.8 (63.5, 76.6)	70.8 (63.5, 76.6)	69.8 (63.9, 76.0)	0.428
Male, N (%)	497 (49)	465 (49)	32 (42)	0.227
Baseline CA19-9, median (IQR)	64.1 (18.1, 314.6)	59.5 (17.7, 292.6)	235.5 (49.8, 693.3)	**<0.001**
Pre-surgery CA19-9, median (IQR)	56.2 (17.4–261.0)	59.5 (17.7, 292.6)	33.0 (15.2–121.6)	0.058
NCCN stage, N (%)				**<0.001**
Resectable	960 (94)	933 (99)	27 (36)	
Borderline	22 (2)	2 (0.2)	20 (26)	
Locally advanced	37 (4)	8 (0.8)	29 (38)	
Operation, N (%)				0.607
Pancreatoduodenectomy	544 (53)	503 (53)	41 (54)	
Distal pancreatectomy	201 (20)	189 (20)	12 (16)	
Total pancreatectomy	274 (27)	251 (27)	23 (30)	
T-stage, N (%)				**0.007**
pT0	1 (1)	0	1 (1)	
pT1	258 (26)	243 (26)	15 (20)	
pT2	276 (27)	246 (26)	30 (39)	
pT3-4	473 (47)	443 (48)	30 (39)	
Unknown	11	11	0	
N stage, N (%)				0.222
pN0	482 (47)	445 (47)	37 (49)	
pN1	284 (28)	258 (27)	26 (34)	
pN2	249 (25)	236 (25)	13 (17)	
Unknown	4	4	0	
Grade of differentiation, N (%)				0.494
Well	101 (11)	98 (11)	3 (7)	
Moderate	550 (58)	526 (59)	24 (55)	
Poor	290 (31)	273 (30)	17 (39)	
Unknown	78	46	32	
R1 margin, N (%)	381 (38)	352 (38)	29 (39)	0.782
Unknown	8	6	2	
Tumor subtype, N (%)				0.285
Tubular	621 (84)	582 (84)	39 (91)	
Colloid	117 (16)	113 (16)	4 (9)	
Unknown	281	248	33	

Bold values are significance of *P* < 0.05.

On postoperative histopathologic review (Table 1), there was no observed difference in the R0-margin rate (38% vs 39%, *P*=0.782) or nodal involvement (pN0: 47% vs 49%, *P*=0.222). However, there was a significant difference in AJCC T-stage with more pT3-4 tumors (48% vs 39%) and less T2 tumors (26% vs 39%) in the upfront surgery group compared with the NAT group (*P*=0.007). There were no significant differences in terms of pancreas-specific complications (*P*=0.474); however, the reoperation rate was higher in the NAT cohort (8% vs 17%, *P*=0.037), while postoperative mortality was similar (2.3% vs 3.8%, *P*=0.361). The administration of adjuvant chemotherapy did not differ between cohorts (NAT: 44% vs upfront surgery: 56%, *P*=0.062). Sixty-one (7%) of patients that underwent upfront surgery, received adjuvant radiation therapy.

### Longitudinal Outcomes

The median follow-up of surviving patients was 43.6 months (IQR: 20.7–79.2). The mOS for those who underwent upfront surgery was 38.0 months (95% CI: 33.7–44.3), which was not statistically different from those who received NAT [27.5 mo (95% CI: 23.1–46.7), *P*=0.121, Fig. 1A]. When adjusted for preoperative variables considered in preoperative treatment decisions (baseline CA19-9 and NCCN stage), NAT had no association with survival [HR: 1.10, (95% CI: 0.57–1.43), *P*=0.672], Fig. 1B). Consequently, also in NCCN-resectable patients only, OS was similar among both groups (mOS upfront surgery: 38.1 mo [95% CI: 34.0–44.4] vs NAT: 35.6 mo [95% CI: 23.1–nr], *P*=0.920, Fig. 2). Moreover, no difference in OS was observed when stratifying NAT patients based on chemotherapy regimen with a mOS in patients treated with FOLFORINOX being 26.2 months (95% CI: 23.1–60.0) compared with 30.9 months (95% CI: 23.8–49.2) in those treated with gemcitabine-based therapy (*P*=0.906).

**FIGURE 1:**
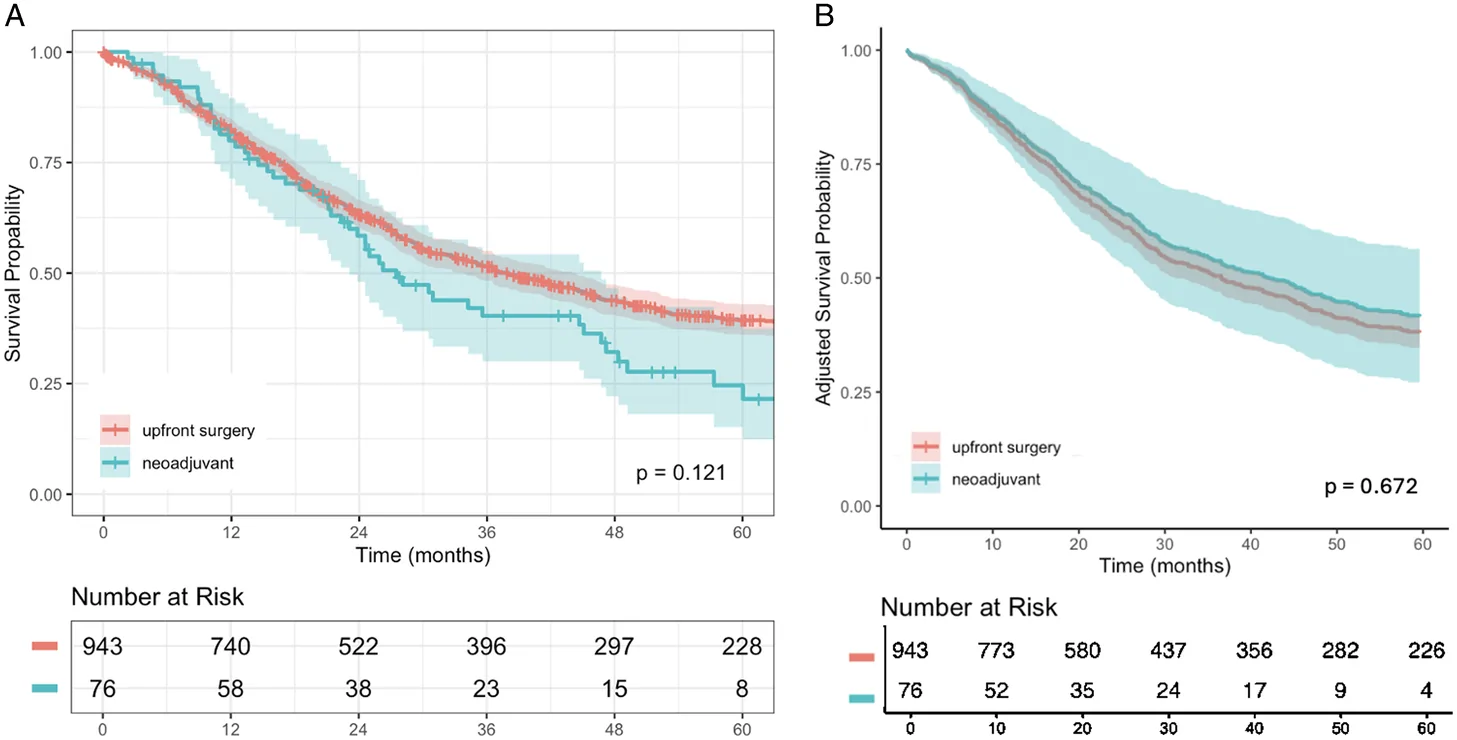
Unadjusted (A) and adjusted (B) Kaplan-Meier curves for overall survival in patients with IPMN-derived pancreatic cancer stratified by neoadjuvant chemotherapy versus upfront surgery.

**FIGURE 2:**
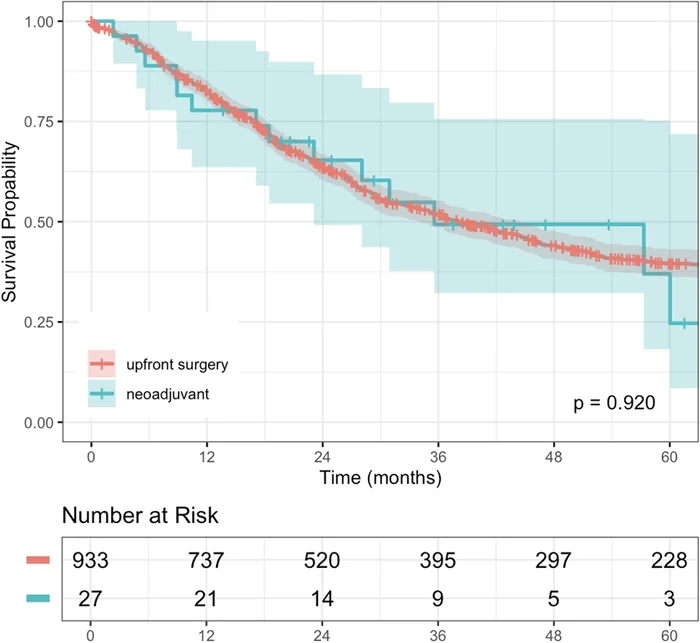
Kaplan-Meier curve for overall survival in patients with resectable IPMN-derived pancreatic cancer stratified by neoadjuvant chemotherapy versus upfront surgery.

Comparing patients who received chemotherapy, those who underwent NAT had worse survival compared with patients who received adjuvant only in the overall cohort [mOS 27.5 mo (95% CI: 27.5–23.1) vs 46.1 mo (95% CI: 39.7–53.1), *P*=0.007]. However, no difference was observed in NCCN-resectable staged patients [mOS neoadjuvant chemotherapy: 35.6 mo (95% CI: 23.1–NR) vs adjuvant chemotherapy only: 46.3 mo (95% CI: 40.2–54.8), *P*=0.410].

### Serologic and Pathologic Treatment Response to NAT

Before NAT, 5 (10%) patients were CA19-9 nonsecretors, while seven (13%) had normal CA19-9 levels and 40 (77%) had elevated CA19-9 (24 patients with missing values). Of those with elevated CA19-9, 18 patients (46%) normalized, and 21 (54%) remained elevated before surgery (1 patient had a missing preoperative CA19-9). After NAT, 32 (48%) patients had a normal CA19-9 level, while 29 (44%) patients had an elevated CA19-9. In CA19-9–producing patients, elevated CA19-9 after NAT was associated with worse OS [mOS: 21.3 mo (95% CI: 17.1–46.7)] as compared with patients who had CA19-9 normalization or were persistently nonelevated CA19-9 [mOS: 45.1 mo (95% CI: 30.5–not reached), *P*=0.017, Fig. 3A).

**FIGURE 3:**
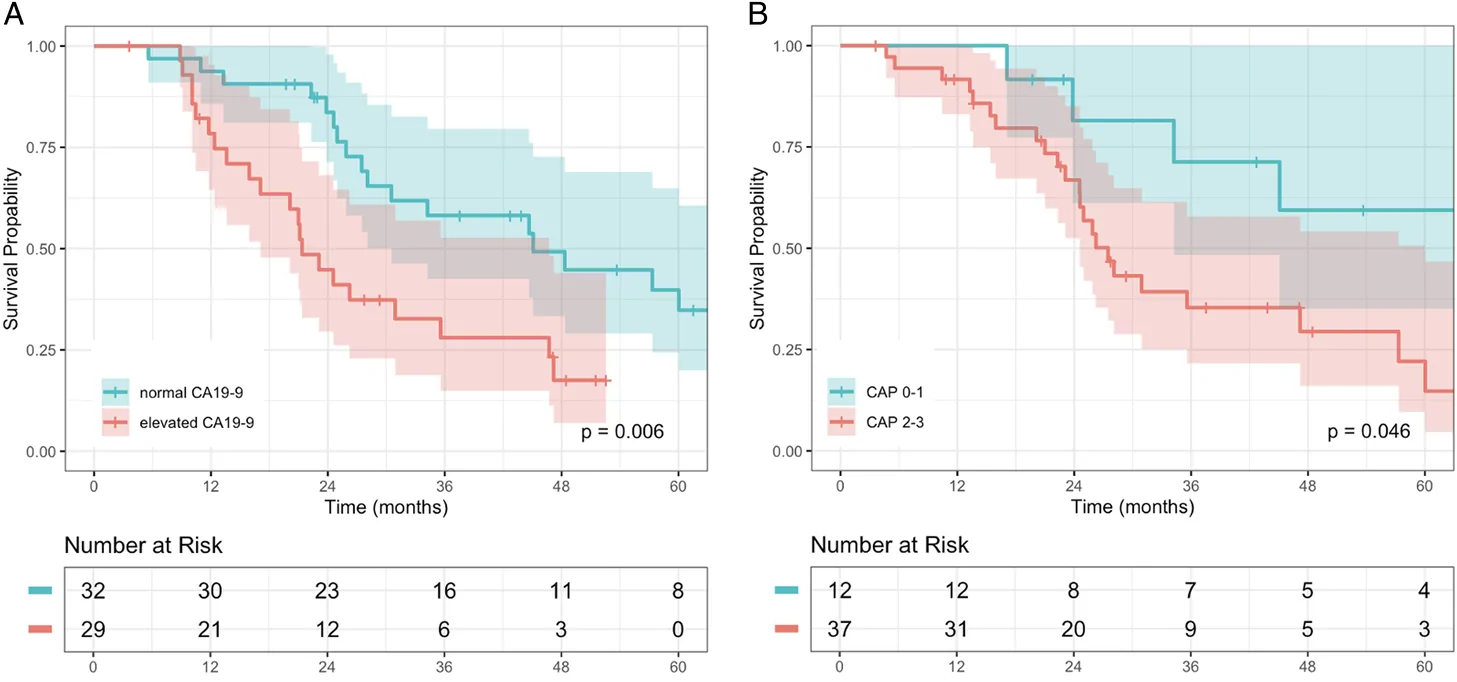
Kaplan-Meier curve for overall survival after neoadjuvant chemotherapy stratified by post-CA19-9 level (A) and CAP score (B).

Of the 49 patients with pathologic response reported, only one (2%) patient had a complete pathologic response, while 11 (22%), 23 (47%), and 14 (29%) patients had marked, partial, or poor response to NAT. The median survival of each group is summarized in Supplement Table 1, Supplemental Digital Content 1, http://links.lww.com/SLA/F429. Those with a major pathologic response were associated with improved OS compared with poor pathologic response [mOS: 63.4 mo (95% CI: 34.2–not reached) versus 27.5 mo (95%CI:24.6–60), *P*=0.046, Fig. 3B].

### Prognostic Factors After Neoadjuvant Treatment

On multivariable Cox regression for OS after NAT, N2 disease [HR: 4.15 (95% CI: 1.71–10.10), *P*=0.002], elevated post-NAT CA19-9 [HR: 2.02 (95% CI: 1.06–3.85), *P*=0.033] and R1 [HR: 2.36 (95% CI: 1.20–4.61), *P*=0.012] emerged as independent factors associated with OS (Fig. 4). Alternatively, after risk adjustment, resectability status according to the NCCN criteria was not associated with overall survival. The C-index for the model is 0.71. For RFS, N2 disease [HR 3.82 (95% CI: 1.65–8.83), *P*=0.002] and R1-status [HR 1.96 (95% CI: 1.05–3.64), *P*=0.034] were associated with shorter RFS in neoadjuvantly treated patients (Supplement Figure 1, Supplemental Digital Content 1, http://links.lww.com/SLA/F429). The C-Index for the RFS model was 0.67.

**FIGURE 4:**
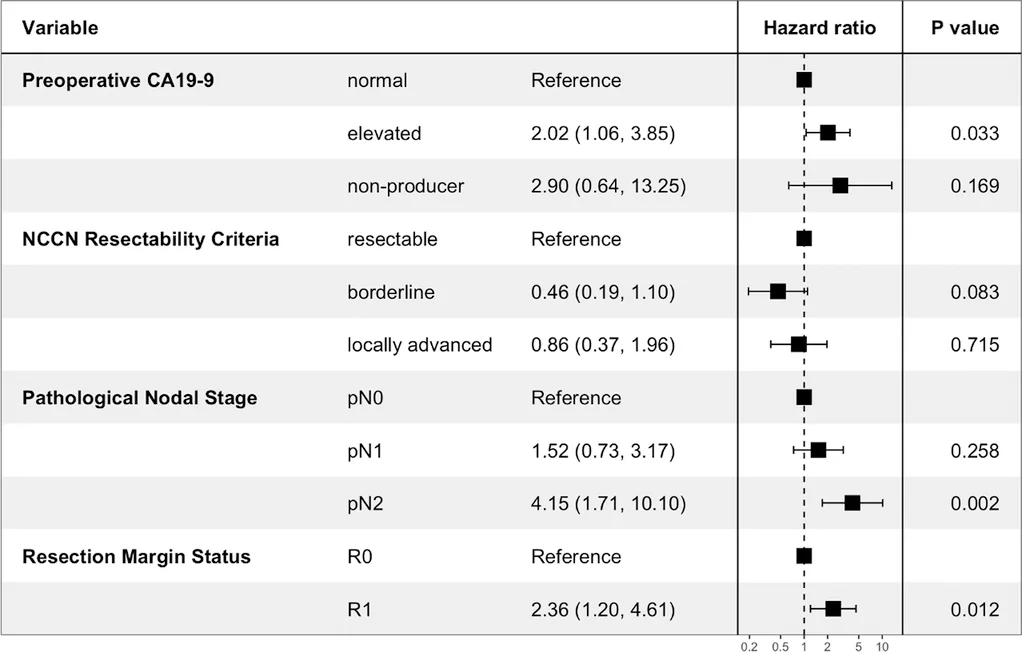
Multivariable Cox regression for overall survival in patients that underwent neoadjuvant chemotherapy.

### Patterns and Timing of Recurrence

With a median follow-up of 40.1 months (IQR: 22.7–53.4 mo) in the NAT cohort, 34 out of 63 patients (54%) with structured follow-up were diagnosed with recurrence. The pattern of recurrence after NAT was predominantly systemic (n=23, 68%). This was similar to the distribution of upfront surgery-treated patients (33% local-only, 67% distant, *P*=0.953) after a median follow-up of 29.6 months (IQR: 14.9–63.2 mo). Although there was no significant difference in recurrence rates (upfront surgery 45% vs NAT 54%, *P*=0.156), the median time from surgery to recurrence was significantly longer after upfront surgery compared with NAT [47.0 mo (37.1–72.3) vs 14.5 mo (11.7–48.1), *P*=0.004].

## DISCUSSION

A neoadjuvant approach in managing PanIN-derived pancreatic cancer is becoming increasingly common, particularly to increase surgical candidacy in patients with BRPC and LAPC.^10,18,19^ This largest and first international multicenter study investigating NAT versus upfront surgery in IPMN-derived pancreatic cancer found that survival outcomes are similar between these 2 cohorts. Furthermore, we observed that CA19-9 dynamics after NAT and histologic pathologic response emerged as significant prognostic factors for biologic response to NAT. Of note, borderline resectable and locally advanced NCCN resectability status was not found to be associated with worse outcomes, possibly indicating that neoadjuvant chemotherapy in these cohorts is effective for patients that make it to surgery, with outcomes comparable to patients with resectable disease.

Like in PanIN-derived PDAC, an improvement in overall survival was not observed when comparing patients who underwent NAT with those who underwent upfront surgery with resectable IPMN-derived pancreatic cancer.^20,21^ Furthermore, we observed that receipt of NAT was more likely in patients with elevated baseline CA19-9 and advanced NCCN resectability status, indicating clear and intentional selection for chemotherapy before resection. Moreover, only four patients that received NAT were known to have colloid histology, supporting the observation of more indolent biology, and hence these patients may have been less likely to be selected for NAT based on resectability and CA19-9.^3^ Similar to in PanIN-derived PDAC, after resection, advanced NCCN resectability status was not associated with worse OS, indicating that NAT may be effective in treating and selecting anatomically and biologically resectable surgical candidates, where those that make it to surgery have similar outcomes to patients with that undergo upfront surgery, most of which have resectable disease.^9,22^ This is consistent with a recent study by Fogliati et al,^11^ who reported NAT in IPMN-derived pancreatic cancer should only be considered in selected patients. Although it is challenging to confidently diagnose an IPMN-derived pancreatic cancer before assessing resected pathology, these results are reassuring in that the effectiveness of NAT for IPMN-derived pancreatic cancer may mirror that observed in PanIN-derived pancreatic cancer.

The administration of NAT provides additional insight into disease biology. Many studies have investigated the prognostic significance and clinical utility of CA19-9 trends during NAT for PanIN-derived PDAC.^23–27^ This is the first study to report on change in CA19-9 after NAT in IPMN-derived pancreatic cancer. Herein, we found that normalization of CA19-9 after NAT was associated with improved survival compared with those who did not have a post-NAT normal CA19-9 level, thus underscoring its prognostic significance of CA19-9 as a biomarker for tumor response in IPMN-derived pancreatic cancer. One international study has already indicated that CA19-9 may provide insight into patients who may require adjuvant chemotherapy after upfront surgery.^7^ Future studies should investigate the role of NAT in the context of both biological and anatomically resectable IPMN-derived pancreatic cancer to inform patient-specific care.^28^ Moreover, the early indication is that FOLFIRINOX and Gemcitabine-based chemotherapy regimens may have similar efficacy in IPMN-derived pancreatic cancer; however, this observation needs to be further investigated.^8^

After resection, differences in pathologic outcomes were observed when comparing patients who underwent NAT to upfront surgery. A sizable number of patients with resected IPMN-derived pancreatic cancer are found to have T1 disease.^15^ Although we did not observe more T1 tumors after NAT, those who received NAT were more likely to have T2 tumors (26% vs 40%) and less likely to have T3-4 tumors (47% vs 40%). This may indicate potential downstaging of larger tumors after NAT as well as an artifact of patient selection. Interestingly, unlike in PanIN-derived PDAC, we did not observe less nodal disease or more R0 margins after NAT.^20,29^ Fogliati et al^11^ also did not observe a reduction in nodal disease and R1 margins after NAT in IPMN-derived pancreatic cancer. One explanation may be that nodal disease, in general, is a rarer outcome in IPMN-derived pancreatic cancer than in PanIN-derived pancreatic cancer, and thus a statistical difference is less likely to be detected. Another explanation may be due to patient selection, given NAT patients tended to have more aggressive disease (elevated CA19-9 and resectability status), thus starting with a higher baseline yield of nodal disease. Larger studies should investigate the impact of NAT histopathologic characteristics, which have been shown to be associated with poorer survival and recurrence.^16,30,31^

A new prognostic variable that emerges after NAT is the pathologic assessment of response.^32^ This study is the first to validate this classification in patients with IPMN-derived pancreatic cancer, where patients with a complete or marked response had significantly longer survival as compared with patients with partial or poor response. Although it remains to be determined if the CAP score employed in PanIN-derived PDAC is the optimal grading scheme for IPMN-derived pancreatic cancer, this study underscores its prognostic value. Employing a precision medicine approach by tailoring preoperative treatment strategies to maximize response may be a promising way to extend survival.

Several limitations should be acknowledged. First, the surgical databases used are highly selected and do not capture patients who do not make it to surgery, thus a subset of patients who undergo NAT but do not make it to surgery due to progression of disease or worsening condition are not included. Prospective trials may partially mitigate this issue if feasible. It is also nearly impossible to accurately diagnose if an invasive component is IPMN-derived on tissue biopsy, thus further limiting our ability to capture IPMN-derived pancreatic cancer patients that progressed on NAT. Moreover, the risk of needle tract seeding in when performing a biopsy of cystic lesions with carcinoma has been described to limit its use in parts of the world which may preclude generalized use of NAT in IPMN-derived pancreatic cancer.^2,33^ Next, although this is the largest study to date on NAT in IPMN-derived pancreatic cancer, future studies are needed with a larger study population to assist in NAT selection and to perform further comprehensive analyses, particularly in different histologic subtypes and for various CA19-9 cutoffs to identify biological responders that may have not normalized. Finally, pathologic evaluation after NAT and reporting practices may differ by institution and year, and unification of international guidelines is ongoing and urgently needed.

In conclusion, neoadjuvant chemotherapy does not confer an associated improvement in overall survival when compared with patients who undergo upfront surgery for resectable IPMN-derived pancreatic cancer. However, after NAT, the emergence of prognostic biomarkers such as CA19-9 dynamics and pathologic response are associated with overall survival in IPMN-derived pancreatic cancer and may provide additional insight into disease biology. After NAT and resection, advanced NCCN resectability was not associated with worse outcomes, indicating that NAT maybe a suitable approach for patients with borderline or locally advanced IPMN-derived pancreatic cancer.

## Supplementary Material
