# Supplementary material for: Neoadjuvant Chemotherapy for Intraductal Papillary Mucinous Neoplasm-derived Pancreatic Cancer
Source: Ann Surg. 2025 Mar 5;284(6):e105–11. doi: 10.1097/SLA.0000000000006687 (PMC13764227; doi:10.1097/SLA.0000000000006687)
Supplement: Supplementary file 1 [file sla-284-e105-s001.docx]

**SUPPLEMENT:**

**Supplement Table 1.** Median overall survival stratified by *Collage of American Pathologists Tumor Response Score

| CAP Score | Median Overall Survival, months (95% CI) |
| --- | --- |
| Complete response | Not reached |
| Marked response | 63.4 (34.2- Not reached) |
| Partial response | 27.5 (23.1- Not reached) |
| Poor response | 26.2 (21.0- Not reached) |

*Collage of American Pathologists Tumor Response Score

**Supplement Figure 1.** Multivariable Cox-regression for recurrence free survival in patients that underwent neoadjuvant chemotherapy
